# Contrast-enhanced ultrasound of both kidneys in healthy, non-anaesthetized cats

**DOI:** 10.1186/s13028-015-0172-5

**Published:** 2015-11-25

**Authors:** Hanna Schweiger, Stefanie Ohlerth, Bernhard Gerber

**Affiliations:** Clinic for Small Animal Internal Medicine, Department for Small Animals, Vetsuisse Faculty, University of Zurich, Zurich, Switzerland; Clinic of Diagnostic Imaging, Department for Small Animals, Vetsuisse Faculty, University of Zurich, Zurich, Switzerland

**Keywords:** Contrast-enhanced ultrasound, Feline renal perfusion, Kidney, Cats, Microbubbles, Buprenorphine

## Abstract

**Background:**

Changes in perfusion are considered to play a key role in the pathophysiology of renal disease. Contrast-enhanced ultrasound (CEUS) has shown a promising diagnostic imaging technique to non-invasively and repetitively quantify tissue perfusion. Examination protocols have varied between studies regarding US equipment, quantification software, the use of sedation or anaesthesia, and animals. The purpose of the present study was, to assess the feasibility of a standardized CEUS protocol for perfusion analysis of both kidneys in nine healthy, non-anaesthetized cats.

**Results:**

CEUS was fairly tolerable for all but one cat. In 6/18 kidneys (2 left, 4 right), a second contrast medium injection was needed due to motion artifacts. Perfusion variables such as peak intensity (PI), wash-in slope (WI_S_), wash-out slope (WO_S_) and mean transit time (MTT) did not significantly differ between left and right renal cortex and medulla nor between the cranial and caudal renal cortex within each kidney. In contrast, for all kidneys, mean PI, WI_S_, and MTT were significantly higher in the cortex than in the medulla (*P* = 0.001, 0.012 and <0.001, respectively).

**Conclusions:**

The herein reported CEUS protocol and the perfusion measurements may serve as a baseline protocol and normal reference values for the evaluation of feline patients. However, the protocol and results may be of limited value in uncooperative animals.

## Background

In patients with suspected renal disease conventional B-mode ultrasound (US) of the kidneys and the entire urinary tract is routinely performed [1]. US represents a non-invasive imaging technique that has no harmful effects on renal function [2]. Nevertheless, ultrasonographic patterns and echogenicity are often non-specific for diffuse renal disease [3] and can be largely inconspicuous even in the case of marked renal dysfunction [4].

Renal function is closely related to organ blood flow and changes in perfusion are considered to play a key role in the pathophysiology of renal disease [5]. For this reason, additional quantitative information about renal perfusion beyond the usual routine diagnostic procedures can be of high diagnostic and prognostic value [6]. Most known methods, like computed tomography, magnetic resonance imaging or scintigraphy, are less appropriate for clinical use due to the need for extensive patient manipulation and the inherent cost of equipment [7]. Moreover, most contrast media currently in use are potentially nephrotoxic and thus contraindicated in cases of acute kidney injury [7]. Plasma clearance methods enable to estimate global organ blood flow only and the renal arterial resistive index derived by pulse-waved Doppler US is considered to be inaccurate [8, 9]. Contrast-enhanced US (CEUS), a diagnostic imaging technique to non-invasively and repetitively quantify tissue perfusion on a capillary level, has been shown promising [10]. CEUS makes use of a contrast medium, which consists of gas-filled microbubbles, that remain strictly in the vasculature [7, 11]. In humans, the administration of a third generation microbubble contrast media (SonoVue^®^) has shown few adverse events [12], does not produce hemodynamic instability and can be used within the critically ill population [13]. This particular contrast medium has also been used in dogs and cats safely [14].

In human medicine, CEUS has already been accepted and recommended for clinical use in a variety of renal pathologies [1]. Perfusion patterns in different regions of the organ can be assessed [7, 10, 15, 16] and with quantitative analysis, changes in renal perfusion may be depicted early in the course of renal allograft nephropathy [16–19], renal artery stenosis [20] or experimentally induced acute kidney injury [21].

So far, several reports documented the use of CEUS in the normal feline kidney [15, 22–24]. However, examination protocols varied between studies regarding US equipment, quantification software, and animals. Most studies included the left kidney only [22–24], and cats were either anaesthetized [15, 22, 23], sedated [15] or unsedated [24]. Anaesthetics significantly influence organ perfusion variables determined with CEUS [10, 25, 26], which has specifically be shown for the feline kidney [24]. Moreover, anaesthesia is not suitable for repeated data collection in critically ill patients. Buprenorphine is a semi-synthetic opioid commonly administered alone to cats, which is primarily used as analgesic and can have an anxiolytic effect [27]. Furthermore, it can be safely used in patients suffering from renal dysfunction [28, 29]. The purpose of the present study was, to assess the feasibility of a standardized CEUS protocol for perfusion analysis of both kidneys in healthy, non-anaesthetized cats administered buprenorphine and an infusion with normal saline.

## Methods

### Animals

Nine cats (8 European Shorthair, 1 British Shorthair) with a median age of 10 months (range, 9 months to 6 years) and a median body weight of 5.4 kg (range, 4.0–6.8 kg) were included. All animals were deemed to be healthy based on physical examination, complete blood count, serum biochemical panel including free T4, urinalysis and aerobic bacterial culture, echocardiography and abdominal ultrasonography. Cystocentesis for urinalysis was carried out following CEUS to exclude iatrogenic ultrasonographic changes and neuronal dependent distortion of the renal blood flow due to irritation of the bladder wall. The study was authorised by the veterinary office of the Canton of Zurich (permit number 26/2014).

### Contrast-enhanced US protocol

Prior to US, animals were fasted for at least 12 h and a 22-gauge indwelling catheter.^1^ was placed in the cephalic vein. Simulating a clinical situation with regard to patients with upper or lower urinary tract obstruction, 0.9 % saline.^2^ was infused intravenously with a rate of 3 ml/kg/h for 2–3 h and stopped shortly before the injection of the contrast medium. In addition, buprenorphine.^3^ at a dose of 20 μg/kg was administered intravenously 1.25–2 h prior to CEUS. After clipping and the application of alcohol and coupling gel, a survey B-mode US of the abdomen was performed. The heart rate and blood pressure were measured.^4^ at the distal front or hind limb at least three times.

CEUS was performed by a radiologist (SO) using a 1–5 MHz broadband curvilinear transducer with pulse inversion.^5^ Settings were kept constant for all cats and optimized during preliminary trials, based on the expertise of the radiologist (oblique ventrodorsal longitudinal plane, dual imaging mode, image depth: 5.5 cm, mechanical index (MI): 0.06, frame rate: 12 Hz, dynamic range: 40, persistence: off, constant time gain compensation and focal zone, low pulse repetition frequency, B-mode gain: 75 %). Cats were in lateral recumbency and manually restricted by 1–2 people. While the transducer remained in the same position, 0.06 ml/kg of a contrast medium.^6^ was injected intravenously as a bolus into a short extension line and rapidly flushed with 5 ml of 0.9 % saline. Simultaneously, images were acquired for approximately 150 s at a rate of one frame per second. CEUS of the left kidney was always performed first. Prior to a subsequent injection, remaining circulating microbubbles were destroyed with the highest MI until there was no subjective evidence of contrast in the kidney, liver, spleen and aorta. Minimum time lag between two contrast injections was 5 min. A maximum of two injections per kidney was performed.

### Contrast-enhanced US analysis

**Fig. 1:**
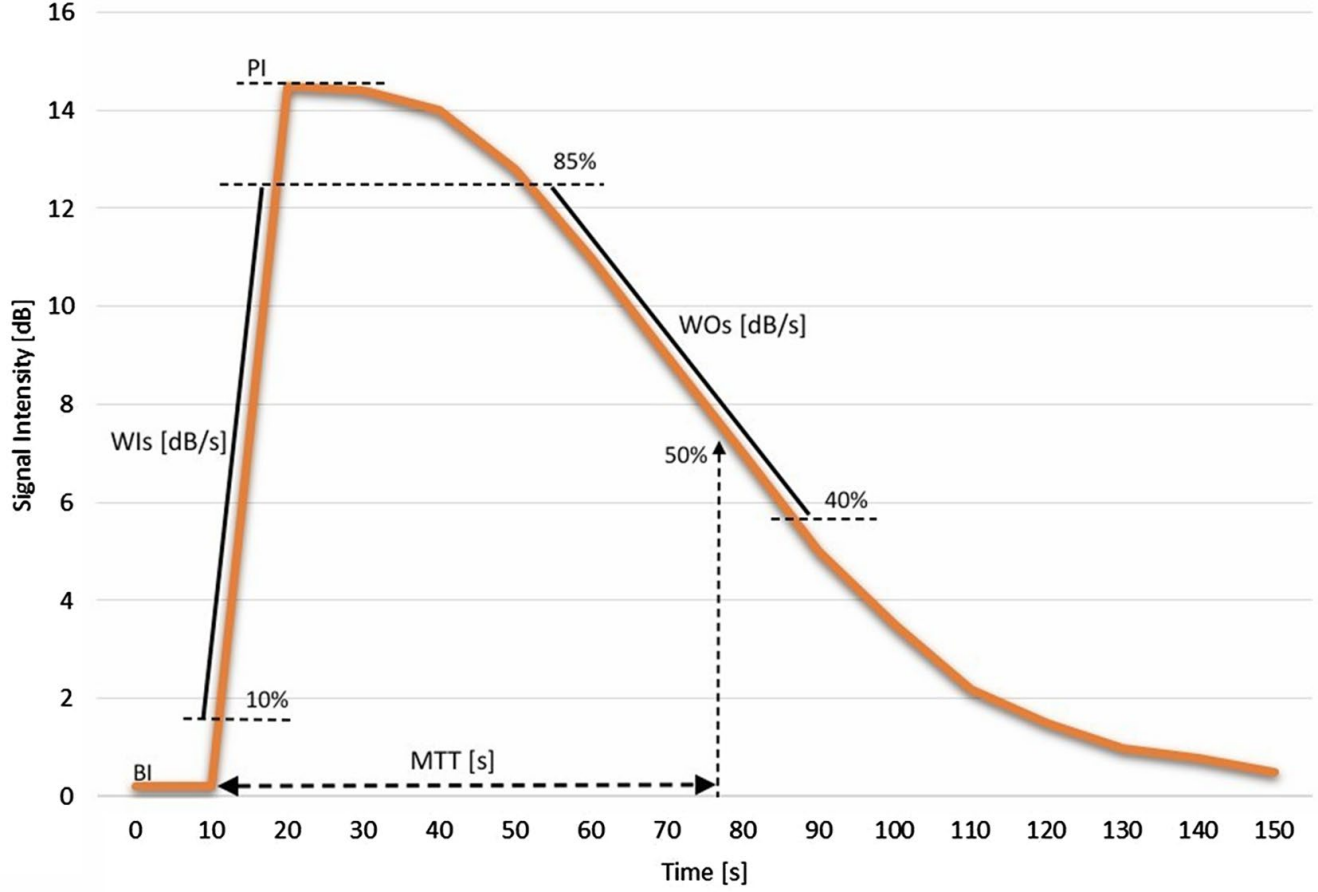
Schematic time intensity curve of a feline kidney. Peak intensity (PI) measured in decibel (dB) was defined as the highest intensity value minus baseline intensity (BI) before the initial rise. Wash-in slope (WI_S_) was calculated with the data 10 % above baseline up to 85 % of the peak value using a linear regression. Wash-out slope (WO_S_) included less data after the peak value (85–40 % of peak intensity value) in order to avoid the exponentially decaying tail. Mean transit time (MTT) was defined as the time from the initial rise until a 50 % decrease of peak intensity [30]

Quantitative analysis was performed by one author (HS) using quantification software.^7^ Automatic and manual motion correction was applied. Regions of interest (ROIs) were always drawn close to the focal zone, where enhancement is most homogeneous [23]. In each cortex, 2 oval-shaped ROIs of a similar size were drawn in the cranial and caudal renal cortex at the same organ depth for a later statistical comparison. Their size ranged from 19.7 to 30.7 mm^2^ (mean 25.1, SD 2.9), and mean organ depth was 29.4 mm (SD 3.0, range 22.6–34.0). In the medulla, a smaller ROI ranging in size from 8.2 to 13.2 mm^2^ (mean 10.9, SD 1.7) was drawn at a mean organ depth of 22.3 mm (SD 3.4, range 17.1–26.6 mm). The medullary ROI was smaller in size to avoid including larger vessels. The following perfusion variables (Fig. 1) were calculated from the raw data using a commercial spreadsheet programme.^8^ Peak intensity (PI) was defined as the highest intensity value minus baseline intensity before the initial rise. Wash-in slope (WI_S_) was calculated with the data 10 % above baseline intensity (BI) up to 85 % of the peak value using a linear regression. Wash-out slope (WO_S_) included less data after the peak value (85–40 % of peak intensity value) in order to avoid the exponentially decaying tail. Mean transit time (MTT) was defined as the time from the initial rise until a 50 % decrease of peak intensity [30].

### Statistical analysis

Statistical analysis was performed using dedicated software.^9^ Association between perfusion variables and systolic blood pressure, heart rate, creatinine values, age or body weight was evaluated with the Pearson’s correlation test. A paired samples t test was used to compare the calculated perfusion variables (PI, WI_S_, WO_S_, MTT) within each kidney (cranial and caudal cortex, cortex and medulla), and between the left and right kidney of each cat. Level of significance was *P* < 0.05.

## Results

### Feasibility

Side effects from contrast media application were not observed in any cat. Systolic blood pressure, heart rate and creatinine values were normal and ranged between 100 and 125 mmHg (mean 115, SD 8.9), 181–218 bpm (mean 195, SD 13.7) and 84–129 μmol/l (mean 101, SD 15.0), respectively. CEUS was fairly tolerated by all but one 9 month-old cat. This animal received 4 bolus injections, however, WO_S_ and MTT could not be determined in the right kidney because of motion artifacts. Four of the 9 cats (44 %) received a total of 3 injections, while the remaining 4 cats (44 %) underwent one CEUS exam per kidney (2 injections in total). Of the 6 repeated injections, 4 were performed in the right kidney. Despite repeated injections, little or no enhancement of one part of the cortex was seen in 3 kidneys due to artifact. Consequently, just 1 ROI was drawn in the cranial cortex of 1 left and the caudal cortex of 2 right kidneys, respectively. Similarly, the renal medulla was only assessable in 13 of 18 (72 %) kidneys (8 left and 5 right) due to breathing or body movement.

### Subjective contrast enhancement

**Fig. 2:**
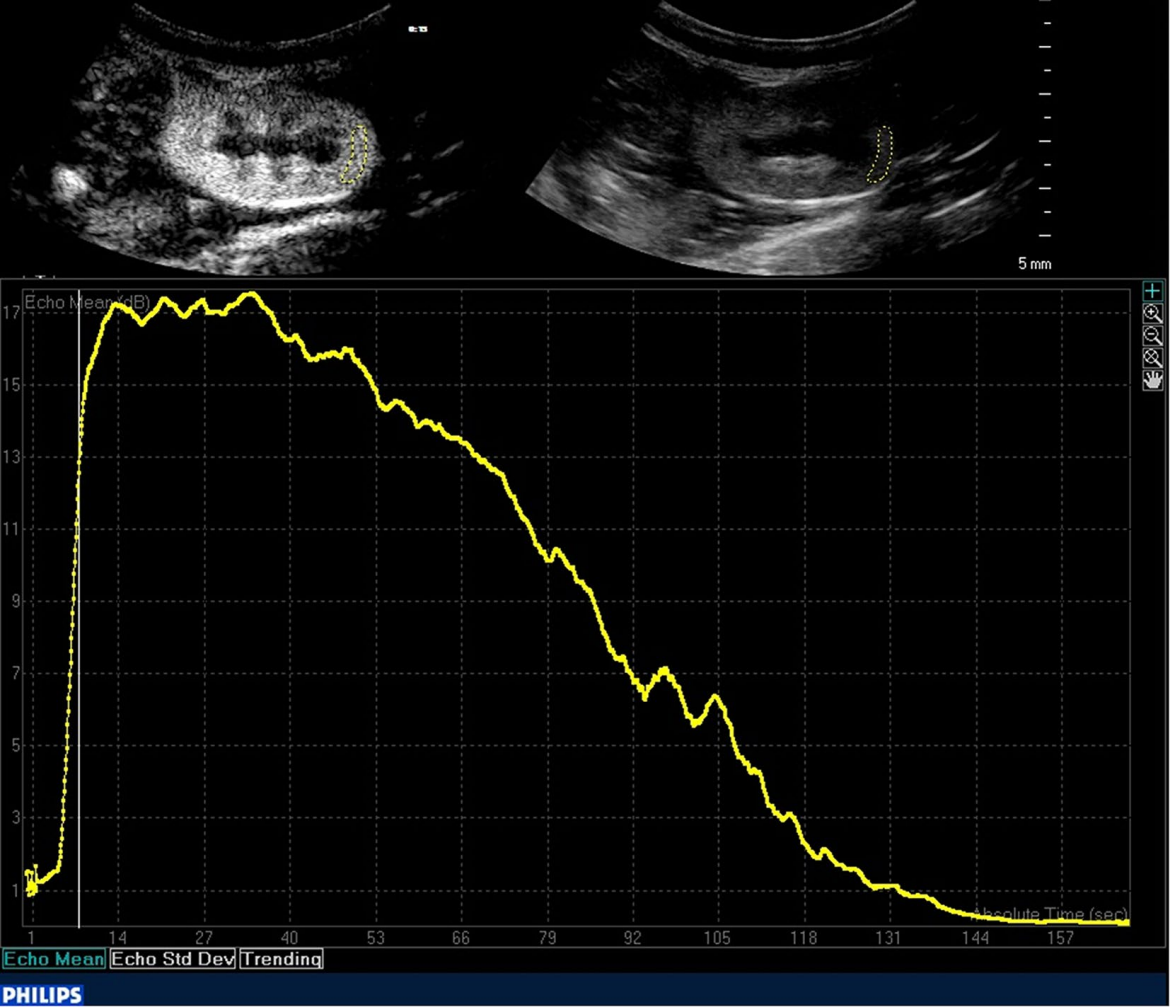
Analysis of contrast enhancement with QLab Software. B-mode (*top right*) and CEUS images (*top left*) of a feline left kidney with a region of interest (ROI) in the caudal cortex (*yellow* *dots*). The corresponding time–intensity curve (*bottom* image) shows a steep initial slope followed by a plateau and gradually descending slope with an exponentially decaying tail

Subjectively, enhancement was first visible at the renal hilus and progressed to the interlobar and arcuate arteries, quickly followed by the renal cortex. TICs of the renal cortex showed a steep initial slope followed by a short plateau after peak intensity and a more gradual decrease with an exponentially decaying tail returning almost to BI (Fig. 2). Compared to the renal cortex, enhancement of the renal medulla occurred later, was less intense with a less steep rise, and faded earlier.

### Quantitative contrast enhancement

#### Comparative analysis of left and right kidneys

The mean and SD of the calculated perfusion variables for the cortex and medulla of the left and right kidneys are shown in Table 1. Perfusion variables did neither correlate with systolic blood pressure, heart rate, creatinine values nor age or body weight. The mean differences between the left and right renal cortex were 3.0 dB for PI (SD 2.0), 0.2 dB/s for WI_s_ (SD 0.1), 0.01 dB/s for WO_s_ (SD 0.01) and 10.7 s for MTT (SD 10.8). For the medulla, mean differences between the left and right kidney were 3.2 dB for PI (SD 2.2), 0.15 dB/s for WI_s_ (SD 0.14), 0.02 dB/s for WO_s_ (SD 0.03) and 10.6 s for MTT (SD 9.1). Mean perfusion values did neither significantly differ between left and right renal cortex and medulla nor between the cranial and caudal renal cortex within each kidney.

**Table 1:** Mean ± SD values of renal CEUS perfusion variables of the left and right kidneys in 9 non-anaesthetized, healthy cats

	Cortex				Medulla			
	Left kidney	n	Right kidney	n	Left kidney	n	Right kidney	n
PI (dB)	14.1 ± 3.0	9 (17)	13.8 ± 3.9	9 (16)	10.0 ± 3.7	8 (8)	12.3 ± 5.6	5 (5)
WI_S_ (dB/s)	1.6 ± 1.3	9 (17)	1.7 ± 1.5	9 (16)	0.5 ± 0.2	8 (8)	0.6 ± 0.2	5 (5)
WO_S_ (dB/s)	−0.16 ± 0.04	9 (17)	−0.15 ± 0.04	8 (14)	−0.15 ± 0.05	8 (8)	−0.16 ± 0.06	5 (5)
MTT (s)	97.6 ± 22.6	9 (17)	98.5 ± 21.2	8 (14)	65.7 ± 13.2	8 (8)	70.3 ± 15.4	5 (5)

No significant differences were found between the left and right kidney (cortex and medulla) for all perfusion variables

*n* number of kidneys (number of ROIs)*, PI* peak intensity, *WI*_*s*_ wash-in slope, *WO*_*s*_ wash-out slope, *MTT* mean transit time

#### Comparative analysis for cortex and medulla

The mean and SD of the calculated perfusion variables for the renal cortex and medulla of all kidneys are shown in Table 2. PI, WI_S_, and MTT were significantly higher in the cortex compared to the medulla (*P* = 0.001, 0.012 and <0.001, respectively).

**Table 2:** Mean ±SD values of renal CEUS perfusion variables in the cortex and medulla of the kidneys in nine non-anaesthetized, healthy cats

	Cortex	n	Medulla	n
PI (dB)^*^	14.0 ± 3.4	18 (33)	10.9 ± 4.4	13 (13)
WI_S_ (dB/s)^*^	1.7 ± 1.4	18 (33)	0.5 ± 0.2	13 (13)
WO_S_ (dB/s)	−0.15 ± 0.04	17 (31)	−0.15 ± 0.05	13 (13)
MTT (s)^*^	98.0 ± 21.6	17 (31)	67.5 ± 13.7	13 (13)

*n* number of kidneys (number of ROIs), *PI* peak intensity, *WI*_*s*_ wash-in slope, *WO*_*s*_ wash-out slope, *MTT* mean transit time

^*^Mean PI, WIS, and MTT were significantly higher in the cortex than in the medulla (P < 0.05)

## Discussion

In the present study, quantitative CEUS perfusion measurements were successfully performed in kidneys of non-anaesthetized cats with the aim of establishing reference values for the used protocol. These results can serve as normal reference values for the evaluation of feline patients. However, cats with suspected chronic renal disease are usually older than the investigated study population, more data from normal cats is needed including various age groups.

Quantitative CEUS perfusion analysis may be affected by various factors such as machine settings, organ depth or signal processing [10, 15, 31]. Therefore, US machine settings should be standardized for every institute, and perfusion values may be only valid in line with a particular protocol. Perfusion values from healthy left feline kidneys were recently published using the same US machine and perfusion computer software [24], however the type of probe was not the same. The present study had an overall higher mean body weight that the previous study and therefore a lower frequency curvilinear transducer had to be used for higher beam penetration.

The subjective renal enhancement pattern observed in the present study is consistent with previous research in the kidneys of healthy dogs [10] and cats [15, 22]. Microbubbles were first imaged in the renal artery and the interlobar arteries followed by a rapid, uniform cortical enhancement ending with a slower and less intense medullary enhancement. Subjective results were supported by the quantitative analysis where PI, WI_S_, and MTT were significantly lower in the medulla compared to the cortex.

Renal perfusion is generally assumed to be bilaterally symmetrical in the absence of renal pathologies or artery stenosis [32]. Using a standardized CEUS protocol, mean perfusion values measured in the present study were indeed not significantly different between related left and right kidneys in all cats, but varied between animals, in particular PI and MTT. The degree of enhancement may vary due to a variable injection time with manual administration of contrast medium. It may also reflect an influence of age, body weight, heart rate or blood pressure. However, in the present study no significant correlations of these variables with perfusion variables were found. WI_S_ and WO_S_ varied less between cats. The Wash-in and wash-out slope do not vary over time and hence, these perfusion variables are least affected by variations in PI [10, 31, 33] and therefore applicable for clinical imaging [23]. However, WI_S_ and WO_S_ can be influenced by mechanical factors such as the saline volume, flushing rate, or the use of a three-way stopcock [31]. The bolus of contrast medium was administered as a preload over a short extension line and flushed by the same volume of saline by one person. Consequently, variation in cortical WI_S_ in the present study most likely reflects the individual physiological changes of the arterial cortical blood flow in cats.

In the present study, CEUS was performed in non-anaesthetized cats, which had received buprenorphine at a recommended clinical dose of 20 μg/kg [34]. Buprenorphine is a semi-synthetic partial mu-receptor opioid agonist with a delayed onset of analgesic action at 30 min and a peak effect at 1.5 h, when administered intravenously. In cats maximal plasma concentration has been detected at 10–45 min [32]. In the present study CEUS of the feline kidneys was conducted when the maximal effect of buprenorphine was expected. Buprenorphine has moderate long-acting (6 h) analgesic properties with few adverse effects [34, 35], minimal respiratory depression and no significant haemodynamic effects [36, 37]. Moreover, buprenorphine can have an anxiolytic effect in animals [27]. Most of the studied cats became more affectionate and were purring. Nonetheless, body movement had to be minimized by manual restriction. Whereas the animals of the present study were young, healthy and agile cats, animals suffering from severe renal dysfunction are seriously or even critically ill patients [38, 39] and for this reason considerably less active. However, that might depend on the cats character, thus the CEUS protocol used in the present study is not recommend for hyperactive or aggressive animals. The premedication with buprenorphine may be of value, in particular, because general anaesthesia is considered to be a contraindication in case of renal disease. Common anaesthetics influence renal function, generally reducing glomerular filtration rate and urine output [40].

Time and effort needed for performing CEUS was minimal in the present study. However, quantification of CEUS perfusion represents a time-consuming post-processing procedure, and therefore, results are not readily available for the clinician.

Motion artifacts caused by the individual were encountered commonly in the present study and gave rise to a second injection in 6/18 kidneys, most frequently in the right kidney (4/6). Although the feline kidneys usually lie more caudal to the rib cage than in dogs [33], possible reasons may be (1) a more cranial position of the right kidney and its proximity to the rib cage causing coupling artifacts, in particular in male cats [41], and (2) the CEUS protocol assessing the right kidney at last and after repositioning the animal.

Due to motion artifacts, quantitative analysis of the medulla was not possible in 5 kidneys. Because of the typical architecture of the renal medulla which is pyramidically separated by interlobar arteries and surrounded by the cortex, size of medullary ROIs is basically smaller than in the cortex and their positioning is more difficult making them more susceptible to organ motion [15]. Variation of perfusion variables caused by ROI size is less than by ROI depth and most affected parameter is the PI [23].

## Conclusions

It was shown that CEUS can be performed successfully in non-anaesthetized cats premedicated with buprenorphine. Perfusion values were determined for the normal left and right renal cortex and medulla. The CEUS protocol and the perfusion measurements can serve as a baseline protocol and normal reference values for the evaluation of feline patients. However, they may be of limited value in uncooperative animals.

## Abbreviations

BI: baseline intensity (dB)
CEUS: contrast-enhanced ultrasound
MI: mechanical index
MTT: mean transit time (s)
PI: peak intensity (dB)
ROI: region of interest
TIC: time intensity curve
US: ultrasonography
WI_S_: wash in slope (dB/s)
WO_S_: wash out slope (dB/s)

## Footnotes

1 VasoVet 22 g, 1 in, 0.9 × 25 mm iv catheter, B. Braun Vet Care, Tuttlingen, DE.

2 NACL Fresenius sol perf 0.9 % 250 ml Freeflex, Fresenius Kabi, Oberdorf, CH.

3 TEMGESIC sol inj 0.3 mg/ml, Reckitt Benckiser, CH.

4 DINAMAP—V100, GE Healthcare, Little Chalfont, UK.

5 iU22, Philips Healthcare, Bothell, Washington, USA.

6 SonoVue^®^, Bracco, Milano, Italy.

7 QLab, Release 8.1.2; Philips Ultrasound, Bothell, WA, USA.

8 Microsoft Excel 2011, Version 14.4.3, Microsoft Corporation, Redmond, WA, USA.

9 SPSS, Version 22.0, IBM, Chicago, Illinois, USA.
